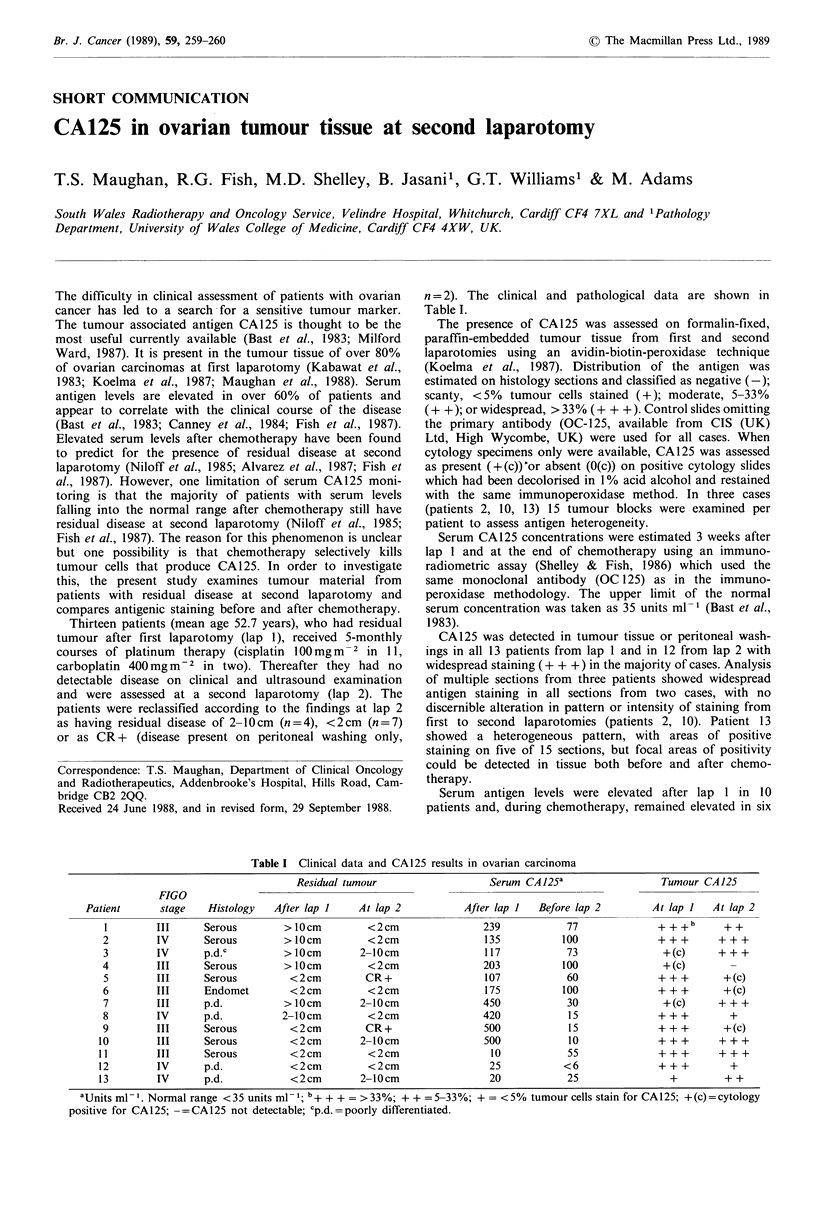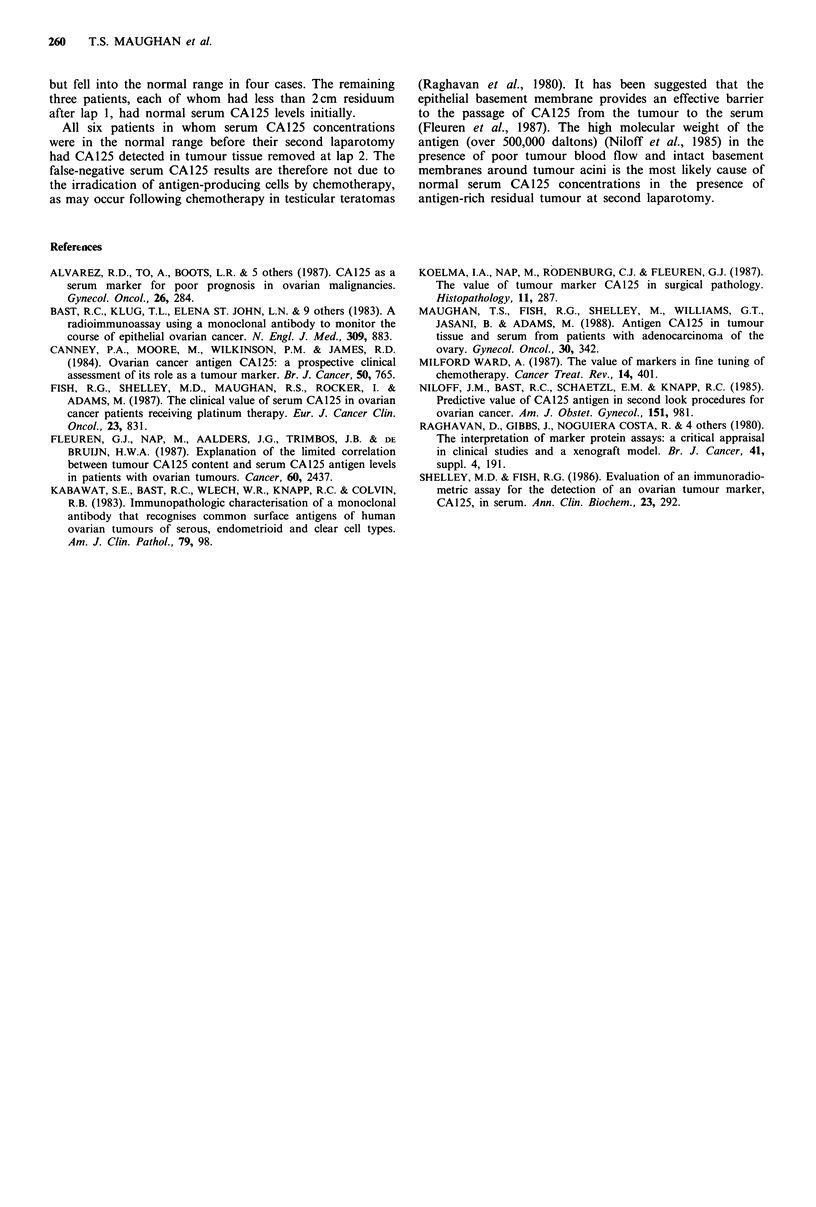# CA125 in ovarian tumour tissue at second laparotomy.

**DOI:** 10.1038/bjc.1989.54

**Published:** 1989-02

**Authors:** T. S. Maughan, R. G. Fish, M. D. Shelley, B. Jasani, G. T. Williams, M. Adams

**Affiliations:** South Wales Radiotherapy and Oncology Service, Velindre Hospital, Whitchurch, Cardiff, UK.


*Br. J. Cancer* (1989), **59**, 259–260 © The Macmillan Press Ltd., 1989

### SHORT COMMUNICATION

### CA125 in ovarian tumour tissue at second laparotomy

T.S. Maughan, R.G. Fish, M.D. Shelley, B. Jasani[1], G.T. Williams[1] & M. Adams

*South Wales Radiotherapy and Oncology Service, Velindre Hospital, Whitchurch, Cardiff CF4 7XL and [1]Pathology Department, University of Wales College of Medicine, Cardiff CF4 4XW, UK.*

The difficulty in clinical assessment of patients with ovarian cancer has led to a search for a sensitive tumour marker. The tumour associated antigen CA125 is thought to be the most useful currently available (Bast *et al.*, 1983; Milford Ward, 1987). It is present in the tumour tissue of over 80% of ovarian carcinomas at first laparotomy (Kabawat *et al.*, 1983; Koelma *et al.*, 1987; Maughan *et al.*, 1988). Serum antigen levels are elevated in over 60% of patients and appear to correlate with the clinical course of the disease (Bast *et al.*, 1983; Canney *et al.*, 1984; Fish *et al.*, 1987). Elevated serum levels after chemotherapy have been found to predict for the presence of residual disease at second laparotomy (Niloff *et al.*, 1985; Alvarez *et al.*, 1987; Fish *et al.*, 1987). However, one limitation of serum CA125 monitoring is that the majority of patients with serum levels falling into the normal range after chemotherapy still have residual disease at second laparotomy (Niloff *et al.*, 1985; Fish *et al.*, 1987). The reason for this phenomenon is unclear but one possibility is that chemotherapy selectively kills tumour cells that produce CA125. In order to investigate this, the present study examines tumour material from patients with residual disease at second laparotomy and compares antigenic staining before and after chemotherapy.

Thirteen patients (mean age 52.7 years), who had residual tumour after first laparotomy (lap 1), received 5-monthly courses of platinum therapy (cisplatin $100\,mg\,m^{-2}$ in 11, carboplatin $400\,mg\,m^{-2}$ in two). Thereafter they had no detectable disease on clinical and ultrasound examination and were assessed at a second laparotomy (lap 2). The patients were reclassified according to the findings at lap 2 as having residual disease of 2–10 cm ($n=4$), <2 cm ($n=7$) or as CR+ (disease present on peritoneal washing only, $n=2$). The clinical and pathological data are shown in Table I.

The presence of CA125 was assessed on formalin-fixed, paraffin-embedded tumour tissue from first and second laparotomies using an avidin-biotin-peroxidase technique (Koelma *et al.*, 1987). Distribution of the antigen was estimated on histology sections and classified as negative (−); scanty, <5% tumour cells stained (+); moderate, 5–33% (+ +); or widespread, >33% (+ + +). Control slides omitting the primary antibody (OC-125, available from CIS (UK) Ltd, High Wycombe, UK) were used for all cases. When cytology specimens only were available, CA125 was assessed as present (+(c)) or absent (0(c)) on positive cytology slides which had been decolorised in 1% acid alcohol and restained with the same immunoperoxidase method. In three cases (patients 2, 10, 13) 15 tumour blocks were examined per patient to assess antigen heterogeneity.

Serum CA125 concentrations were estimated 3 weeks after lap 1 and at the end of chemotherapy using an immunoradiometric assay (Shelley & Fish, 1986) which used the same monoclonal antibody (OC 125) as in the immunoperoxidase methodology. The upper limit of the normal serum concentration was taken as 35 units $ml^{-1}$ (Bast *et al.*, 1983).

CA125 was detected in tumour tissue or peritoneal washings in all 13 patients from lap 1 and in 12 from lap 2 with widespread staining (+ + +) in the majority of cases. Analysis of multiple sections from three patients showed widespread antigen staining in all sections from two cases, with no discernible alteration in pattern or intensity of staining from first to second laparotomies (patients 2, 10). Patient 13 showed a heterogeneous pattern, with areas of positive staining on five of 15 sections, but focal areas of positivity could be detected in tissue both before and after chemotherapy.

Serum antigen levels were elevated after lap 1 in 10 patients and, during chemotherapy, remained elevated in six

Correspondence: T.S. Maughan, Department of Clinical Oncology and Radiotherapeutics, Addenbrooke's Hospital, Hills Road, Cambridge CB2 2QQ.
Received 24 June 1988, and in revised form, 29 September 1988.

**Table I** Clinical data and CA125 results in ovarian carcinoma

| Patient | FIGO stage | Histology | Residual tumour: After lap 1 | Residual tumour: At lap 2 | Serum CA125[a]: After lap 1 | Serum CA125[a]: Before lap 2 | Tumour CA125: At lap 1 | Tumour CA125: At lap 2 |
|---|---|---|---|---|---|---|---|---|
| 1 | III | Serous | >10 cm | <2 cm | 239 | 77 | + + +[b] | + + |
| 2 | IV | Serous | >10 cm | <2 cm | 135 | 100 | + + + | + + + |
| 3 | IV | p.d.[c] | >10 cm | 2–10 cm | 117 | 73 | +(c) | + + + |
| 4 | III | Serous | >10 cm | <2 cm | 203 | 100 | +(c) | − |
| 5 | III | Serous | <2 cm | CR+ | 107 | 60 | + + + | +(c) |
| 6 | III | Endomet | <2 cm | <2 cm | 175 | 100 | + + + | +(c) |
| 7 | III | p.d. | >10 cm | 2–10 cm | 450 | 30 | +(c) | + + + |
| 8 | IV | p.d. | 2–10 cm | <2 cm | 420 | 15 | + + + | + |
| 9 | III | Serous | <2 cm | CR+ | 500 | 15 | + + + | +(c) |
| 10 | III | Serous | <2 cm | 2–10 cm | 500 | 10 | + + + | + + + |
| 11 | III | Serous | <2 cm | <2 cm | 10 | 55 | + + + | + + + |
| 12 | IV | p.d. | <2 cm | <2 cm | 25 | <6 | + + + | + |
| 13 | IV | p.d. | <2 cm | 2–10 cm | 20 | 25 | + | + + |

[a]Units $ml^{-1}$. Normal range <35 units $ml^{-1}$; [b]+ + + = >33%; + + = 5–33%; + = <5% tumour cells stain for CA125; +(c) = cytology positive for CA125; − = CA125 not detectable; [c]p.d. = poorly differentiated.

but fell into the normal range in four cases. The remaining three patients, each of whom had less than 2 cm residuum after lap 1, had normal serum CA125 levels initially.

All six patients in whom serum CA125 concentrations were in the normal range before their second laparotomy had CA125 detected in tumour tissue removed at lap 2. The false-negative serum CA125 results are therefore not due to the irradication of antigen-producing cells by chemotherapy, as may occur following chemotherapy in testicular teratomas (Raghavan *et al.*, 1980). It has been suggested that the epithelial basement membrane provides an effective barrier to the passage of CA125 from the tumour to the serum (Fleuren *et al.*, 1987). The high molecular weight of the antigen (over 500,000 daltons) (Niloff *et al.*, 1985) in the presence of poor tumour blood flow and intact basement membranes around tumour acini is the most likely cause of normal serum CA125 concentrations in the presence of antigen-rich residual tumour at second laparotomy.

### References

ALVAREZ, R.D., TO, A., BOOTS, L.R. & 5 others (1987). CA125 as a serum marker for poor prognosis in ovarian malignancies. *Gynecol. Oncol.*, **26**, 284.

BAST, R.C., KLUG, T.L., ELENA ST. JOHN, L.N. & 9 others (1983). A radioimmunoassay using a monoclonal antibody to monitor the course of epithelial ovarian cancer. *N. Engl. J. Med.*, **309**, 883.

CANNEY, P.A., MOORE, M., WILKINSON, P.M. & JAMES, R.D. (1984). Ovarian cancer antigen CA125: a prospective clinical assessment of its role as a tumour marker. *Br. J. Cancer*, **50**, 765.

FISH, R.G., SHELLEY, M.D., MAUGHAN, R.S., ROCKER, I. & ADAMS, M. (1987). The clinical value of serum CA125 in ovarian cancer patients receiving platinum therapy. *Eur. J. Cancer Clin. Oncol.*, **23**, 831.

FLEUREN, G.J., NAP, M., AALDERS, J.G., TRIMBOS, J.B. & DE BRUIJN, H.W.A. (1987). Explanation of the limited correlation between tumour CA125 content and serum CA125 antigen levels in patients with ovarian tumours. *Cancer*, **60**, 2437.

KABAWAT, S.E., BAST, R.C., WLECH, W.R., KNAPP, R.C. & COLVIN, R.B. (1983). Immunopathologic characterisation of a monoclonal antibody that recognises common surface antigens of human ovarian tumours of serous, endometrioid and clear cell types. *Am. J. Clin. Pathol.*, **79**, 98.

KOELMA, I.A., NAP, M., RODENBURG, C.J. & FLEUREN, G.J. (1987). The value of tumour marker CA125 in surgical pathology. *Histopathology*, **11**, 287.

MAUGHAN, T.S., FISH, R.G., SHELLEY, M., WILLIAMS, G.T., JASANI, B. & ADAMS, M. (1988). Antigen CA125 in tumour tissue and serum from patients with adenocarcinoma of the ovary. *Gynecol. Oncol.*, **30**, 342.

MILFORD WARD, A. (1987). The value of markers in fine tuning of chemotherapy. *Cancer Treat. Rev.*, **14**, 401.

NILOFF, J.M., BAST, R.C., SCHAETZL, E.M. & KNAPP, R.C. (1985). Predictive value of CA125 antigen in second look procedures for ovarian cancer. *Am. J. Obstet. Gynecol.*, **151**, 981.

RAGHAVAN, D., GIBBS, J., NOGUIERA COSTA, R. & 4 others (1980). The interpretation of marker protein assays: a critical appraisal in clinical studies and a xenograft model. *Br. J. Cancer*, **41**, suppl. 4, 191.

SHELLEY, M.D. & FISH, R.G. (1986). Evaluation of an immunoradiometric assay for the detection of an ovarian tumour marker, CA125, in serum. *Ann. Clin. Biochem.*, **23**, 292.